# Oncolytic adenoviruses in bladder and kidney cancers: emerging strategies and next frontiers

**DOI:** 10.3389/fmicb.2025.1729087

**Published:** 2025-12-09

**Authors:** Jia Yao, Dmitry M. Shayakhmetov

**Affiliations:** 1 Departments of Pediatrics and Medicine, Lowance Center for Human Immunology, Emory University School of Medicine, Atlanta, GA, United States; 2 Winship Cancer Institute of Emory University, Atlanta, GA, United States; 3 Emory Vaccine Center, Emory University School of Medicine, Atlanta, GA, United States

**Keywords:** oncolytic adenovirus, combination therapy, immunomodulation, kidney cancer, bladder cancer, clinical trial

## Abstract

Engineering oncolytic adenoviruses (OAds) to treat renal and bladder cancers has advanced rapidly. Leveraging insights into adenovirus biology, the molecular pathology of cancer, and recent progress in tumor immunology, multiple strategies have been developed to enhance the antitumor specificity and overall efficacy of OAds as anticancer therapeutics. Combination with other cancer treatment modalities has shown synergistic effects, further augmenting their therapeutic potential. In this review, we discuss the most recent advances in oncolytic adenovirus designs that tailor this vector platform for the treatment of renal and bladder cancers. We further summarize findings from preclinical animal studies demonstrating tumor suppression by OAds, as well as results from clinical trials evaluating their safety and efficacy in cancer patients. Collectively, the accumulated data highlights the significant potential of engineered oncolytic adenoviruses as a novel therapeutic modality for cancer patients that have limited treatment options.

## 1 Introduction

Oncolytic viral therapy has emerged as a prominent approach in cancer treatment. Genetically engineered oncolytic viruses (OVs) eradicate tumors through direct tumor cell killing while sparing normal cells and by inducing anti-tumor immunity, turning “immunologically cold” tumor microenvironments into “hot” ones (Du et al., 2025; Muthukutty and Yoo, 2023). To date, both DNA and RNA viruses have been adapted for the development of oncolytic therapies. In DNA virus category, adenovirus, herpes simplex virus (HSV), and vaccinia virus have been tested for the treatment of various tumors. Notably, the HSV-based IMLYGIC^®^ (talimogene laherparepvec) became the first US FDA-approved oncolytic viral therapy for advanced melanoma (Vázquez-Arreguín et al., 2025). Various RNA viruses, including reovirus, vesicular stomatitis virus (VSV), Newcastle disease virus (NDV), measles virus, poliovirus, coxsackievirus A21, and Seneca Valley virus, have been investigated for their anti-tumor effects (Lundstrom, 2022). While monotherapy with oncolytic viruses may not achieve optimal efficacy, combination therapies with other anti-tumor approaches such as chemotherapy, radiotherapy, immune checkpoint inhibitors (ICIs), chimeric antigen receptor T cells (CAR-T cells), and other targeted therapies have significantly improved their effectiveness (Du et al., 2025; Muthukutty and Yoo, 2023; Zou et al., 2023).

Adenoviruses (Advs) were first discovered in adenoid tissue back in 1950's and therefore received the name “Adenovirus” Hilleman and Werner, (1954). These non-enveloped, double-stranded DNA viruses have a broad tissue tropism across various vertebrate hosts, including humans. Owing to their ability to infect both dividing and non-dividing cells, along with advancements in genome manipulation and capsid engineering, adenoviruses are now extensively utilized as vectors in gene therapy Kozarsky and Wilson, (1993) and vaccine development (Tatsis and Ertl, 2004); (Coughlan et al., 2022). The most recent highlight probably is its utilization in the COVID-19 vaccine development (Halperin et al., 2022); (Klingler et al., 2024); (Logunov et al., 2020). Except for gene and vaccine delivery, adenoviruses have also been developed as oncolytic viruses due to their potent lytic activity, tumor selectivity, strong immunogenicity, capacity to express exogenous therapeutic genes and compatibility with combination therapies (Dobbelstein, 2004). From the early demonstration of the E1B 55K–mutant dl1520 (a.k.a. ONYX-015), which selectively replicated in and killed p53-deficient human tumor cells (Bischoff et al., 1996), to the recent development of Ad5-3M virus that evades host innate immunity, rendering safe and efficient systemic cancer therapy (Atasheva et al., 2020), oncolytic adenoviruses (OAds) have consistently shown promising anti-cancer potentials. In this review, we summarize recent advances in the application of OAds for the treatment of kidney and bladder cancers and elaborate on their therapeutic mechanisms, clinical progress, and future perspectives.

## 2 Unmet needs in the treatment of kidney and bladder cancers

**Figure 1:**
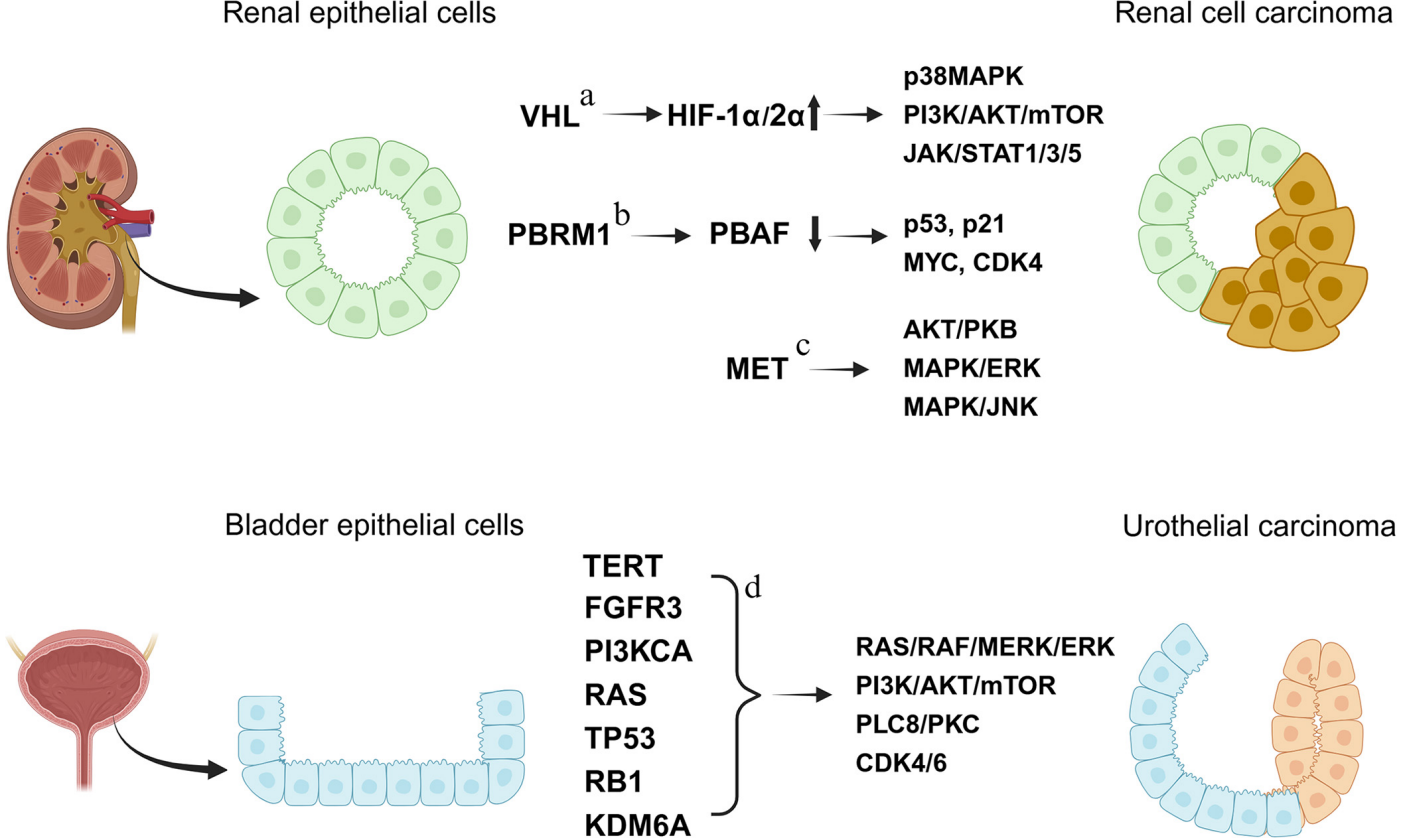
Major genetic mutations and dysregulated signaling pathways in renal and bladder cancers. VHL, von hippel–lindau; MAPK, mitogen-activated protein kinase; PI3K, phosphoinositide 3-kinase; mTOR, mechanistic target of rapamycin; JAK, janus kinase; STAT, signal transducer and activator of transcription; PBRM1, polybromo 1; PBAF, polybromo-associated BAF complex; MYC, myc proto-oncogene, bHLH transcription factor; CDK4/6, cyclin-dependent kinase 4/6; MET, MET proto-oncogene, receptor tyrosine kinase; ERK, extracellular signal–regulated kinase; JNK, c-jun N-terminal kinase; FGFR3, fibroblast growth factor receptor 3; RAS, rat sarcoma viral oncogene homolog; KDM6A, lysine demethylase 6A. The figure was created with BioRender.com. References included: a-Baldewijns et al. (2010) and Shirole and Kaelin (2023); b-Brugarolas (2013), Cai et al. (2019), and Varela et al. (2011); c-Pal et al. (2018), Rhoades Smith and Bilen (2019), and Linehan et al. (2016); d-Hayashi et al. (2020), Qiu et al. (2023), and Hurst and Knowles (2022).

Urological cancers originate in the organs and tissues of the urinary tract and male reproductive system, encompassing malignancies of the kidney, bladder, prostate, urethra, penis, and testicles. The etiology of renal and bladder cancers involves multiple genetic driver mutations. This leads to dysregulated signaling pathways that promote tumor growth and progression (Figure 1). Bladder cancer is the most common type of urinary tract cancers, makes up about 50% of all urinary tract malignancies. The American Cancer Society estimates that there will be 84,870 new cases of bladder cancer, and 17,420 deaths from bladder cancer in 2025 (https://www.cancer.org/cancer/types/bladder-cancer.html). Kidney cancer, with 85% of the cases being renal cell carcinoma (RCC), accounts for 4–5% of all cancers, and 2.3% of all cancer deaths in the United States (https://www.cancer.org/cancer/types/kidney-cancer/about/key-statistics.html). As with all cancers, achieving the best possible outcomes in the treatment of these cancers relies on early detection and diagnosis. However, kidney and bladder cancers are often asymptomatic in the early stages, making early detection and diagnosis challenging. The prognosis for stage IV kidney or bladder cancer is poor, the 5-year survival rate is less than 10%.

The classical treatments for kidney cancer are surgical resection, and the less invasive tumor ablation approaches to remove the tumor mass. In the past 20 years, with the unraveling of deeper molecular mechanisms of kidney cancer pathology, the discovery of tumor biomarkers, and the deciphering of the tumor immunological environment landscape, targeted therapies such as vascular endothelial growth factor (VEGF) and mechanistic target of Rapamycin (mTOR) signaling inhibitors, as well as immune checkpoint inhibitors (ICIs), have been incorporated into kidney cancer management (Yang et al., 2023). Combination therapies with ICIs and VEGF-targeted agents have demonstrated improved therapeutic outcomes and are now widely used as first-line treatments for advanced or metastatic RCC.

For bladder cancer, which is predominantly urothelial carcinoma, platinum-based chemotherapy has remained the standard first-line therapy for many decades (Liu et al., 2025; Rani et al., 2023). Similar to kidney cancer, newer approaches such as ICIs, antibody-drug conjugates (ADCs), and targeted agents have shown notable survival benefits. Nevertheless, their effectiveness can vary considerably depending on the molecular characteristics of the tumor cells (Hashimoto et al., 2025).

Despite these advances in the treatment of kidney cancer and bladder cancer, new therapies to further improve therapeutic outcomes are still in urgent need, especially for locally advanced and metastatic cases. In a US-based study, analysis of more than 8,100 patients with advanced or metastatic urothelial cell carcinoma revealed that more than 25% of these patients did not receive first-line systemic therapy. For those treated, less than 25% received immuno-oncology-based monotherapy. Patients without systemic treatment options had median overall survival (OS) and progression-free survival (PFS) of only 6–7 months, highlighting a substantial unmet need for more effective treatments (Geynisman et al., 2022).

The limited therapeutic success of new targeted agents and immune therapies in bladder and kidney cancers, particularly in advanced or metastatic cases, stems from their pronounced intratumoral heterogeneity, adaptive resistance mechanisms, and ability to escape immune surveillance. Researchers analyzed over 1,000 scientific publications from 64 countries/regions reporting on targeted therapy resistance in metastatic renal cell carcinoma (mRCC). The bibliometric analysis of these studies understored the pivotal influence of the tumor microenvironment (TME) in driving resistance to targeted therapies in RCC (Wang et al., 2025). The analysis further identified cancer stem cells and tumor cells exhibiting epithelial-mesenchymal transition (EMT) as key contributors to the therapeutic resistance (Wang et al., 2025). A recent review summarized the mechanisms underlying immunotherapy resistance in genitourinary cancers (Evans et al., 2024). It emphasized the roles of genetic abnormalities or immune system dysregulation, dynamic changes in the levels of growth factors, cytokines, and the TME.

**Figure 2:**
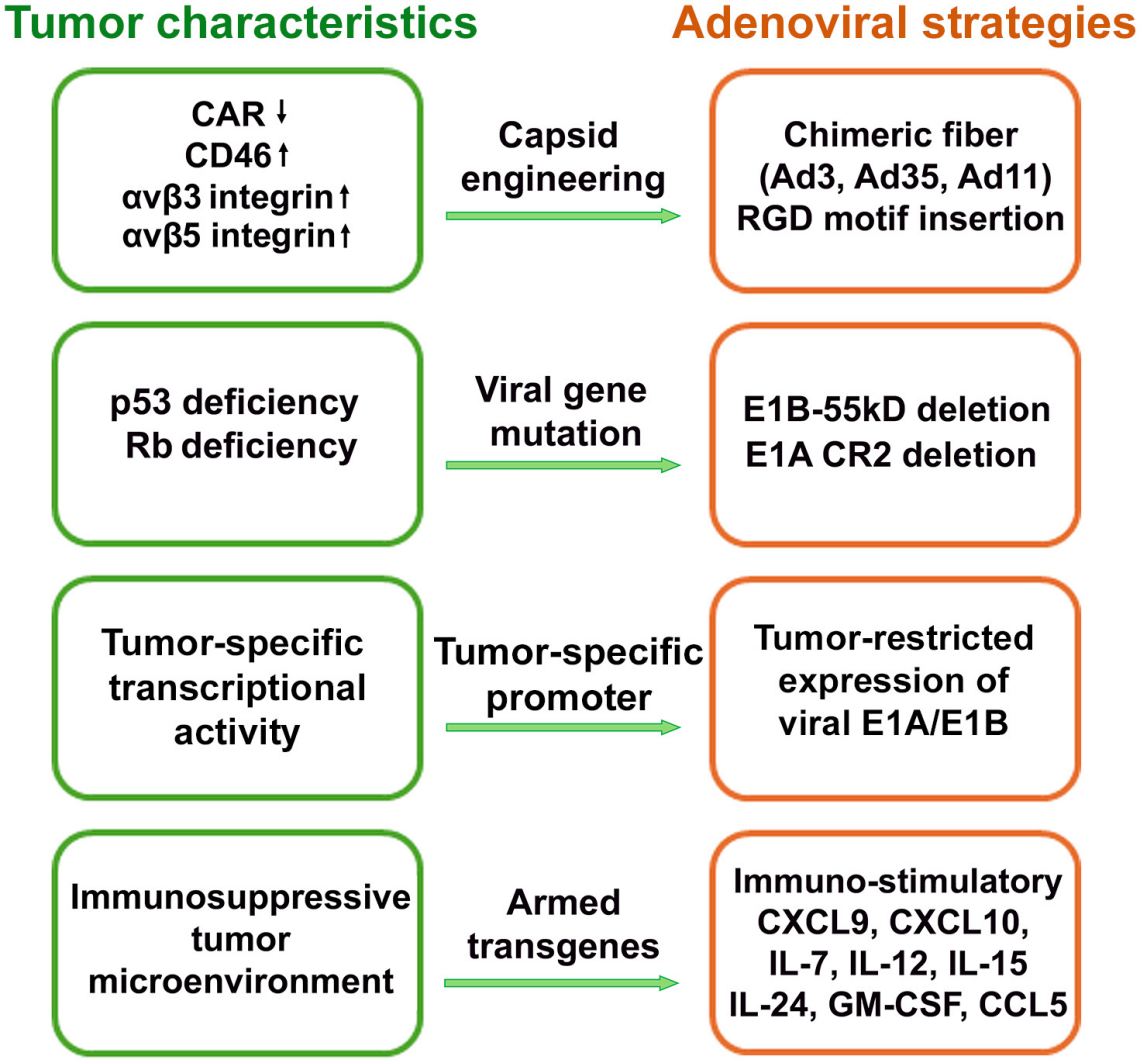
Oncolytic adenovirus design strategies based on key molecular, cellular, and microenvironmental features of renal and bladder cancers.

The unique anti-tumor properties of oncolytic viruses make them well-suited to overcoming key treatment barriers in kidney and bladder cancers. Over the past two decades, oncolytic viruses based on HSV-1, vaccinia virus, reovirus, adenovirus, Sendai virus, and Newcastle disease virus have been explored in preclinical studies for the treatment of kidney and bladder cancers, with several being tested in clinical trials (Taguchi et al., 2017). In the following sections, we will discuss the advances in application of oncolytic adenoviruses (OAds) in the treatment of kidney and bladder cancer. The key molecular and cellular characteristics of renal and bladder cancers have been exploited to design OAds with tumor-selective activity (Figure 2). Table 1 summarizes strategies for developing OAds for renal and bladder cancer treatment, with details discussed in the following sections.

**Table 1:** Oncolytic adenovirus engineering strategies for renal and bladder cancers.

**OAds**	**Capsid modifications**	**Genome modifications**	**Armed transgenes**	**Cancers**	**References**
Ad5F35/Mkp-E1	Ad35 fiber knob	Midkine promoter driving E1		Renal	Nagaya et al., 2012
F5/35-ZD55-hep27	Ad35 fiber shaft, knob	E1B-55KD deletion	Hep27	Renal	Fang et al., 2016
CRAd-synNotch	Ad35 fiber knob	COX-2 promoter driving E1	synNotch (CD44-N-HIF3α4)	Bladder	Ueki et al., 2025
Ad5/3-Δ24	Ad3 fiber knob	Δ24 deletion in E1A gene		Renal	Guse et al., 2007
Ad5-Δ24RGD	RGD motif in HI-loop	Δ24 deletion in E1A gene		Renal	Guse et al., 2007
TILT-517 (Ad5/3-E2F-d24-hIL7)	Ad3 fiber knob	E2F promoter driving E1A, Δ24 deletion in E1A gene, Δ145 deletion in E1B-19K	human IL-7	Renal	Arias et al., 2025
AxdAdB3-F/RGD	RGD motif in HI-loop	mutation in CR2 region of E1A, E1B-55kD deletion		Bladder	Wang et al., 2014a
Onco^Ad^.RGD-hTERT-TRAIL	RGD motif in HI-loop	Δ24 deletion in E1A gene, E1B-55kD deletion, hTERT promoter driving E1A	TRAIL	Bladder	Yang et al., 2015
Ki67-ZD55-IL-24		Ki67 promoter driving E1A, E1B-55kD deletion	IL-24	Renal	Chen et al., 2015
G250-ZD55-Ki67		G250 promoter driving E1A, E1B-55kD deletion	Ki67-shRNA	Renal	Liu et al., 2012
AxdAdB-3		mutation in CR2 region of E1A, E1B-55kD deletion		Bladder	Wang et al., 2006
OBP-301		hTERT promoter driving E1A and E1B (linked by IRES)		Renal	Huang et al., 2010
rAd.sT		hTERT promoter driving E1A	sTGFβRIIFc	Renal	Yang et al., 2019
Ad9xHRE1A		9xHRE promoter driving E1A, E2F-1 promoter driving E4		Renal	Cuevas et al., 2003
HRE-Ki67-Decorin		HRE-Ki67 promoter driving E1A	Decorin	Renal	Zhang et al., 2020
AdLCY		E1B-55kD deletion, 6xHRE-9xOct4RE-CMV mini promoter driving E1A		Bladder	Lu et al., 2015
PPE3-SEA	Ad11 fiber knob	hTERT promoter driving E1A, HRE-miniCMV promoter driving E1B	SEA	Bladder	Han et al., 2013
CG8840		UPII promoter driving E1A and E1B		Bladder	Zhang et al., 2002
Ad/PSCAE/UPII/E1A-AR		PSCAE-UPII promoter driving E1A-androgen receptor chimeric protein		Bladder	Zhai et al., 2012
Ad.9OC		E1B-55kD deletion, 9xOct3/4RE-miniCMV promoter driving E1		Bladder	Wu et al., 2008
Ad-CAIX^promoter^-AIM2		CAIX promoter driving E1A	AIM2	Renal	Chai et al., 2020
Ad5/35E1apsurvivinE4	Ad35 fiber knob	Survivin promoter driving E1A and E4, most of E3 deletion		Bladder	Seo et al., 2014
KD01		E1A CR2 deletion, E3 ADP deletion	tBID (in ADP location)	Bladder	Guo et al., 2025
ZD55-Ki67		E1B-55kD deletion,	Ki67-shRNA	Renal	Zheng et al., 2009
Ki67-ZXC2-double siRNA		Ki67 promoter driving E1A	Ki67-shRNA, hTERT-shRNA	Renal	Fang et al., 2014
Ad-hTERT-HSV-TK			hTERT promoter driving HSK-TK	Renal	Tian et al., 2013
HY-oAd			scmIL-12, mGM-CSF, and relaxin	Bladder	Yoon et al., 2024
Ad5/3-CXCL9, Ad5/3-CXCL10, Ad5/3-IL-15	Ad3 fiber knob	Δ24 deletion in E1A gene	CXCL9 or CXCL10 or IL-15	Renal	Feodoroff et al., 2024
Ad5/F11p-PSCAE-UPII-E1A	Ad11p fiber shaft and knob	PSCAE-UPII promoter driving E1A		Bladder	Cao et al., 2017
Ad/PSCAE/UPII/E1A		PSCAE-UPII promoter driving E1A		Bladder	Wang et al., 2014b; Zhang et al., 2017
Ad-VT (Ad-hTERT-E1a-Apoptin)		hTERT promoter driving E1A	Apoptin	Bladder	Shang et al., 2021
Xvir-N-31	RGD motif in HI-loop	Deletion of 13S splice acceptor and most of CR3 of E1A, deletion of E1B-19K		Bladder	Hindupur et al., 2020; Lichtenegger et al., 2019
Adv-CRB3			CRB3 and GM-CSF	Bladder	Zhang et al., 2024b
OAV-Decorin		Ki67 promoter driving E1A	Decorin	Renal	Zhang et al., 2022
Ad5-ZD55-CCL5-IL12		E1B-55kD deletion	CCL5 and IL-12	Renal	Fang et al., 2023
H101		E1B-55kD deletion, partial deletion in E3		Bladder	Hua, 2022
CG0070 (Cretostimogene grenadenorepvec)		E2F-1 promoter driving E1A, gp19KD deletion in E3	GM-CSF	Bladder	Ramesh et al., 2006
Ad-HOC-E1		Osteocalcin promoter driving E1A and E1B		Renal	Hsiao et al., 2012

COX-2, cyclooxygenase-2; hTERT, human telomerase reverse transcriptase; IRES, internal ribosome entry site; HRE, hypoxia response element; UPII, uroplakin II; PSCAE, prostate stem cell antigen enhancer; CAIX, carbonic anhydrase IX; SEA, staphylococcus enterotoxin A; HSK-TK, herpes simplex virus thymidine kinase; AIM2, absent in melanoma 2; tBID, truncated BH3 interacting domain death agonist; TRAIL, TNF-related apoptosis-inducing ligand; GM-CSF, granulocyte–macrophage colony-stimulating factor; CXCL9, C-X-C motif chemokine ligand 9; CXCL10, C-X-C motif chemokine ligand 10; CRB3, crumbs homolog 3; CCL5, C-C motif chemokine ligand 5; IL-7, interleukin-7; IL-12, interleukin-12; IL-15, interleukin-15; IL-24, interleukin-24; shRNA, short hairpin RNA; Oct3/4, octamer transcription factor-3/4.

## 3 Strategies to enhance kidney and bladder tumor specificity of oncolytic adenoviruses

### 3.1 Capsid engineering of adenoviruses to enhance targeted transduction of kidney and bladder tumors

Achieving tumor specific infection, replication and killing is central in developing OAds. For OAds to effectively eradicate kidney and bladder tumors, they must selectively infect and replicate within malignant cells. Several strategies of capsid modification have been developed to enhance specificity of tumor cell infection while sparing healthy normal cells intact. To date, more than 100 unique human adenovirus serotypes have been discovered, and even more non-human adenovirus serotypes exist. This large adenovirus virome creates ample opportunities for constructing adenovirus variants with chimeric capsid proteins, where individual components of the virus can be substituted for homologous components of alternate serotypes with desirable cell and tissue transduction properties. Adenoviral infection begins with attachment and internalization through interactions with cell surface receptors. Because the fiber knob domain of the adenovirus mediates receptor binding, modifying this region to recognize alternative, tumor-associated receptors represents a straightforward strategy to improve infection efficiency in these cancers. The most widely used Ad5-based vectors rely on the coxsackievirus and adenovirus receptor (CAR), which is often absent or downregulated in kidney and bladder carcinomas (Nagaya et al., 2012; Fang et al., 2016; van der Poel et al., 2002; Gotoh et al., 2013). In contrast, renal cell carcinomas (RCCs) express high levels of CD46 (Nagaya et al., 2012; Fang et al., 2016; Guse et al., 2007) and desmoglein-2 (DSG2), the primary cellular receptors for several subgroup B adenoviruses, most notably Ad35 (Gaggar et al., 2003) and Ad3 (Wang et al., 2011). This receptor profile provides a unique advantage for harnessing subgroup B adenovirus entry over traditional Ad5-based platform, as these receptors are abundantly expressed on RCC and bladder tumor cells but limited in normal tissue, enabling more selective infection and stronger oncolytic potency. Accordingly, substituting the Ad5 fiber knob with that of Ad35 (Nagaya et al., 2012; Fang et al., 2016; Ueki et al., 2025) or Ad3 (Guse et al., 2007; Arias et al., 2025) has been shown to markedly enhance the tumor specificity and cytolytic activity of engineered OAds *in vitro*, as well as in RCC or muscle—invasive bladder cancer (MIBC) xenograft and Syrian hamster models.

Additional capsid engineering strategies have been employed to improve tumor selectivity, such as inserting the RGD-4C motif into the adenovirus fiber. This modification retargets viral entry toward αvβ3/5 integrins, which are commonly upregulated in malignant cells. The RGD-modified adenovirus AxdAdB3-F/RGD demonstrated no toxicity in normal bladder mucosa-derived cell line, while inducing potent cytotoxic effects in both CAR-positive and CAR-negative bladder cancer cell lines. Furthermore, intravesical administration of this OAd in an orthotopic model of CAR-deficient human bladder cancer suppressed tumor progression and prolonged survival (Wang et al., 2014a). Similarly, insertion of RGD motif in the HI loop of fiber exhibited increased infection ability and cytotoxic effect of Onco^Ad^.RGD-hTERT-EGFP in CAR-negative bladder cancer cells compared to parental virus Onco^Ad^.hTERT-EGFP (Yang et al., 2015).

Capsid modifications, including receptor retargeting and introduction of integrin-binding motifs, significantly enhance the tumor specificity, infectivity, and cytolytic activity of OAds in kidney and bladder cancers. These engineering strategies enable effective targeting of CAR-deficient renal and bladder tumors and provide a strong rationale for further therapeutic development of OAds.

### 3.2 Modification of viral essential genes to restrict replication of oncolytic adenoviruses to kidney and bladder cancer cells

The early development of tumor-specific OAds takes advantage of the virus's ability to hijack cellular p53 and retinoblastoma tumor suppressor (Rb) pathways for its replication. The E1A proteins, which are the first viral proteins synthesized upon infection, function by binding Rb, p300, and other cellular proteins to drive cells into S phase, thereby creating an environment favorable for viral replication. The Δ24 adenovirus was engineered to have a 24-base pair deletion (corresponding to eight amino acids) in the Rb-binding domain of E1A, preventing the formation of the E1A/Rb complex. As a result, the virus is unable to replicate in cells with intact Rb function but can selectively replicate in and kill Rb-deficient tumor cells, such as gliomas (Fueyo et al., 2000). The E1B-55k viral protein binds and inactivates cellular p53 protein therefore normal anti-virus function of p53 is comprised and virus could replicate. Early generation of OAd-dl1520 lacking E1B-55k expression exhibits 100 times more replication efficiency in p53-deficenct tumor cells compared to normal cells (Bischoff et al., 1996). Mutations in p53 and Rb pathways have been implicated in the pathogenesis of kidney and bladder cancers (Miyamoto et al., 1995; Knudsen et al., 2020; Amendolare et al., 2022; van Rhijn et al., 2004; Hayashi et al., 2020). Consequently, E1B-55K deletion and Δ24 modifications have been widely utilized in the construction of OAds for bladder and kidney cancers (Fang et al., 2016; Guse et al., 2007; Yang et al., 2015; Chen et al., 2015; Liu et al., 2012; Wang et al., 2006).

### 3.3 Utilization of tumor-selective promoters for driving oncolytic adenovirus replication in kidney and bladder cancer cells

Another strategy to achieve cancer cell-restricted replication of OAds is to place essential viral genes such as E1A and E1B under the control of tumor-specific promoters. Many genes involved in cell cycle regulation, proliferation, apoptosis, and survival are frequently mutated and mostly upregulated in cancer cells, making their promoters useful for selective targeting. This transcriptional control enables tumor-specific viral replication while reducing toxicity in normal tissues.

The human telomerase reverse transcriptase (hTERT) promoter is a widely studied tumor-specific regulatory element for OAd development. Both kidney and bladder cancers exhibit significantly higher hTERT expression compared with normal tissues (Morozov et al., 2021). In bladder cancer, hTERT detection has clinical significance: FDA-approved immunocytochemistry (ICC) in urine cytology detects hTERT for diagnosis and monitoring. In a cohort of 337 cytology samples, hTERT ICC positivity was found highly correlated with the malignancies (about 96–100% accuracy), while 96.7% of benign cases were hTERT-negative (Moon et al., 2023). Harnessing the hTERT promoter to regulate tumor-specific viral replication offers the benefit of maximizing anti-tumor efficacy while minimizing damage to normal tissues in renal and bladder cancers. OBP-301, an OAd with hTERT-driven E1A/E1B, showed strong anti-tumor activity in orthotopic and metastatic RCC mouse models, the anti-tumor effects were further enhanced by IL-2 co-administration (Huang et al., 2010). Similarly, rAd.sT used the hTERT promoter to restrict E1A expression in immunocompetent breast and renal cancer models (Yang et al., 2019). In the previously discussed Onco^Ad^.RGD-hTERT-EGFP/TRAIL (Yang et al., 2015), the hTERT promoter was employed to drive E1A expression in addition to incorporating a fiber-RGD modification along with Δ24 and E1B-55K deletions. This engineered virus demonstrated strong cytotoxic activity against bladder cancer- initiating cells.

A common feature of solid tumors, including kidney and bladder cancers, is hypoxia. Hypoxia-inducible factor (HIF) is a key driver of renal tumorigenesis and is also associated with resistance to chemotherapy and radiotherapy. In clear cell renal cell carcinoma (ccRCC), the most common and highly vascularized subtype of RCC, HIF becomes constitutively activated due to loss of function of the von Hippel-Lindau (VHL) tumor suppressor gene. Mechanistically, VHL is a component of an E3 ubiquitin ligase complex that mediates HIF-α subunits degradation under normoxic conditions. When VHL is inactivated, HIF-α escapes degradation, accumulates, and activates a transcriptional program that includes pro-tumorigenic factors such as vascular endothelial growth factor (VEGF; Baldewijns et al., 2010). A dual-regulated Ad9xHRE1A virus, in which the essential viral E4 gene is driven by the E2F-1 promoter and the E1A gene is controlled by an artificial promoter containing nine tandem hypoxia-responsive elements (HREs) for HIF binding, achieved specific anti-tumor effects in VHL-deficient ccRCC (Cuevas et al., 2003). Another example of such regulation is a chimeric promoter composed of the Ki67 promoter positioned upstream of an HRE sequence to drive E1A expression; the resulting OAd HRE-Ki67-Decorin virus suppressed tumor growth in subcutaneous RCC models (Zhang et al., 2020). Using HRE elements to control E1A expression, OAds such as AdLCY (Lu et al., 2015) and PPE3-SEA (Han et al., 2013) also demonstrated great anti-tumor efficacy in bladder cancer models. These studies highlight the broader applicability of HIF/HRE-driven transcriptional control for tumor-selective adenoviral replication beyond kidney cancer.

The urothelium-specific promoters are particularly useful for bladder cancer–specific targeting. Segments of the uroplakin II (UPII) promoter preferentially drive expression in urothelial carcinoma cells. Building on this, OAd-CG8840, in which E1A and E1B are regulated by the UPII promoter, efficiently infected and lysed bladder carcinoma cells *in vitro* and induced significant regression of tumor xenografts in mice, particularly when combined with docetaxel (Zhang et al., 2002). Another group used the UPII promoter to drive an E1A-androgen receptor (E1A-AR) fusion protein expression, where intratumoral injection of Ad/PSCAE/UPII/E1A-AR significantly reduced bladder tumor growth in subcutaneous mouse models (Zhai et al., 2012).

Other tumor-specific promoters that have been harnessed to drive activity of OAds in renal and bladder cancers include the stem-cell-associated marker Oct3/4 (Lu et al., 2015; Wu et al., 2008), cyclooxygenases-2 (COX-2; Ueki et al., 2025), midkine (Nagaya et al., 2012), the renal cancer-associated antigen G250 / carbonic anhydrase IX (CAIX; Liu et al., 2012; Chai et al., 2020), the tumor proliferation marker Ki67 (Zhang et al., 2020) and survivin (Seo et al., 2014). These tumor-selective regulatory elements expand the repertoire of transcriptional targeting strategies, thereby improving both the specificity and safety of OAd therapy in urological malignancies.

## 4 Arming oncolytic adenoviruses with therapeutic transgenes

### 4.1 Therapeutic transgenes to augment tumor cell death

Oncolytic adenoviral vectors can accommodate a payload up to ~3 kb, making it a useful platform for incorporating therapeutic genes. This feature thereby expands the strategies available to enhance the anti-tumor efficacy of OAds through incorporation of various transgenes.

Beyond the intrinsic lytic activity of these viruses, inducing other forms of tumor cell death represents a rational therapeutic strategy. Several studies demonstrate that arming OAds with pro-apoptotic genes can strengthen their therapeutic potency. For example, expression of hep27 within a fiber-chimeric vector (F5/35-ZD55-hep27) activated the Hep27-MDM2-p53 axis, leading to enhanced apoptosis and superior tumor control in renal carcinoma models compared to the parental virus (Fang et al., 2016). Similarly, incorporation of TRAIL, a selective activator of death receptor-mediated apoptosis, into Onco^Ad^.RGD-hTERT yielded Onco^Ad^.RGD-hTERT-TRAIL, which suppressed growth of bladder cancer-initiating cells and inhibited xenograft progression (Yang et al., 2015). Other pro-apoptotic transgenes include truncated BID (tBID) and AIM2 (Chai et al., 2020). Infection with the KD01 virus specifically induced tBID expression in bladder cancer cells, causing mitochondrial pore formation and subsequent apoptosis (Guo et al., 2025).

Another direction involves knockdown tumor-specific proliferation markers to induce cell death. Ki67 expression is tightly linked to tumor progression and poor prognosis, its promoter has been employed to drive selective OAd replication in renal cancer cells. Arming the OAds with shRNA-cassette knocking down Ki67 further reduced renal cancer cell growth by suppressing this proliferation marker and triggering apoptosis (Liu et al., 2012; Zhang et al., 2020; Zheng et al., 2009; Fang et al., 2014).

Beyond apoptosis, suicide gene strategies such as Ad-hTERT-HSV-TK/GCV (Ganciclovir) have shown efficacy in RCC by combining tumor-specific replication with the well-established HSV-TK/GCV system. In renal cell carcinoma mouse models, this approach reduced tumor burden and extended survival, underscoring the therapeutic value of coupling OAds with clinically validated cytotoxic systems (Tian et al., 2013).

Taken together, these examples illustrate that arming OAds with apoptotic inducers, proliferation suppressors, or suicide gene systems represent complementary approaches to achieve the goal of broadening the scope of intrinsic oncolysis.

### 4.2 Expressing transgenes to enhance anti-tumor immune responses and remodel tumor microenvironment

Mobilizing the body's immunity to combat tumors is not new in urological cancer therapy; intravesical instillation of Bacillus Calmette-Guérin (BCG) was pioneered more than 50 years ago to treat non-muscle invasive bladder cancer (NMIBC; Morales et al., 1976). One of the two major anti-tumor mechanisms of OAds is induction of innate and adaptive immunity to suppress tumor cell growth. The OAds mediated tumor cell lysis leads to release of viral progeny, pathogen- and damage-associated molecular patterns (PAMPs and DAMPs), and tumor-associated neoantigens into the tumor microenvironment (TME). These molecules stimulate the generation of proinflammatory cytokines and chemokines by innate immune cells. The resulting inflammatory milieu also enhances antigen presentation and recruits cytotoxic T lymphocytes in the tumor tissue, converting the “immunologically cold” TME to “hot” and promoting systemic anti-tumor immune responses. Therefore, arming OAds with transgenes encoding molecules that stimulate immune responses or remodel TME could further augment their anti-tumor efficacy.

Transforming growth factor-β (TGF-β) plays multiple roles in physiological conditions. It functions as a pro-tumorigenic factor in cancer development. Activation of TGF-β signaling stimulates cancer cell proliferation and evasion of immune surveillance. In addition, TGF-β is thought to promote metastases by driving cytoskeletal changes that underlie the epithelial-to-mesenchymal transition (EMT) and to model TME as an immunosuppressive factor. Elevated TGF-β expression strongly predicts a worse prognosis in bladder cancer (Kianmehr et al., 2024); TGF-β/Smad signaling pathway exerts pro-oncogenic and pro-metastatic function in RCC (Dias et al., 2025), rationally, the TGF-β pathway is considered as a therapeutic target and has been harnessed in OAds for renal and bladder cancer therapy. The rAd.sT was engineered to express a soluble TGF receptor II fused with human IgG Fc fragment (sTGFβRIIFc) to inhibit TGF-β signaling. Infection of both human and mouse renal tumor cells with rAd.sT produced high levels of sTGFβRIIFc protein and induced cytotoxicity. However, only weak replication was observed in mouse renal cancer cells, suggesting that mechanisms other than viral replication or direct oncolysis contributed to cytotoxicity in these tumors (Yang et al., 2019). Intratumoral injection of rAd.sT inhibited mouse Renca xenograft tumor growth in immunocompetent mice. Importantly, intratumoral injection of rAd.sT increased CD8^+^ T lymphocytes and CD4^+^ T memory cells in the peripheral blood, pointing to a direct anti-tumor immunological effect by suppressing TGF-β signaling. Beyond targeting TGF-β, OAds have been armed with other immune-modulating molecules. Increased recruitment of CD3, CD4, CD8 T cells, and NK cells to the spleen or bladder/renal tumor tissue has also been achieved by arming OAds with the superantigen Staphylococcus enterotoxin A (SEA; Han et al., 2013), with IL-12, GM-CSF, and relaxin (Yoon et al., 2024), or with CXCL9, CXCL10, and IL-15 (Feodoroff et al., 2024).

Notably, advanced OAd approaches for renal and bladder cancers often integrate all the strategies described above—capsid modification, tumor-restricted promoter utilization, and therapeutic transgene expression—to achieve the most favorable outcomes.

## 5 Combination therapies with oncolytic adenoviruses for kidney and bladder cancers

The pathological mechanisms of development of kidney and bladder cancers are complicated, involving both intrinsic genetic mutations and environmental factors. Multiple anti-tumor modalities have been used to treat these malignancies based on different mechanisms of carcinogenesis and metastasis. The advances are represented by the recently developed targeted therapies, immunotherapies, and oncolytic viral therapies. Monotherapy with a single anti-tumor therapeutic usually does not achieve the best outcomes due to the complicated biological characteristics of the tumor, leading to the adoption of combination therapy.

### 5.1 Oncolytic adenoviruses complement conventional tumor therapies in renal and bladder cancers

The three pillars of conventional cancer treatments—surgery, chemotherapy, and radiotherapy, remain in the regimens of kidney and bladder cancer treatments, though each approach has disadvantages regarding specificity and long-term benefit. Surgery removes the bulk of tumor tissue but can leave residual tumor cells and miss micrometastases, thus resulting in recurrence and metastasis. OAds can complement surgery by directly lysing tumor cells and enhancing the anti-tumor immune response to eliminate metastatic cancer cells. While this strategy has not been reported in renal and bladder cancer treatments, its clinical exploration appears warranted. Chemotherapy is often associated with systemic toxicity and may foster drug resistance. Especially for RCC, chemotherapy is not effective due to the expression of drug-efflux pumps in the renal-origin cells. However, combination therapy with OAds and the chemotherapy reagent cisplatin has been investigated by several groups for bladder cancer. The anti-tumor efficacy of bladder-cancer-specific OAds: Ad5/F11p-PSCA-UPII-E1A (Cao et al., 2017), Ad/PSCAF/UPII/E1A (Wang et al., 2014b), and KD01 (Guo et al., 2025), was tested using bladder cancer cell lines as monotherapy or in combination with cisplatin. All studies showed synergistic anti-tumor effects in combination treatment, with increased tumor cell apoptosis appearing to be the mechanism. Since cisplatin is an approved drug for treating advanced bladder cancer, this combination approach holds promise for a clinical translation.

RCC is traditionally considered resistant to radiotherapy. With the advancements in radiation delivery technology, the radiotherapy becomes feasible in RCC treatment (Christensen and Hannan, 2022). Radiation has been approved to increase cytotoxicity of Ki67-ZD55-IL-24 virus in RCC cells via mitochondrial apoptotic cell death (Chen et al., 2015). Similar effects were observed when radiation was combined with a bladder-cancer-specific Ad-PSCAE-UPII-E1A virus to kill bladder cancer cells (Zhang et al., 2017).

In the era of rapid advances in novel cancer treatments, the conventional cancer therapies retain their value in renal and bladder cancers management through strategic integration with OAds.

### 5.2 Augmenting targeted therapies with oncolytic adenoviruses in kidney and bladder cancers

Carcinogenesis of renal and bladder cancers involves dysregulation of multiple signaling pathways. Aberrant signaling in the JAK/STAT, AMPK/Raptor/mTOR, PI3K/AKT/mTOR, VHL/HIF/VEGFR/mTOR, FGFR3/MYC, and Wnt/β-catenin pathways has often been observed during the progression of renal cell carcinoma and urothelial carcinoma. Unlike traditional chemotherapy drugs that kill rapidly dividing cells, small-molecule inhibitors and antibody-drug conjugates (ADCs) target specific proteins and pathways. Several such agents have been approved by the FDA for clinical use in kidney and bladder cancer treatment. Efficacy of monotherapy with targeted drugs is greatly influenced by patients' genetic background. The heterogeneity of tumors renders monotherapy unsuccessful and leads to resistance, whereas combination therapy can address this limitation. The anti-tumor efficacy of OAds combined with targeted drugs has been evaluated. AD-VT (Ad-hTERT-E1a-apoptin) specifically replicates in and induces death of bladder cancer cells. The anti-tumor mechanism of AD-VT is attributed to autophagic cancer cell death through AMPK/Raptor/mTOR signaling. Its therapeutic activity is further improved when combined with Rapamycin—an inhibitor of mTOR (Shang et al., 2021).

The STAT3/5 inhibitor Stattic, but not JAK1/2 inhibitors, reduced proliferation of more than 20 bladder cancer cell lines. Pre-treatment of T24 and UMUC-3 bladder tumor cells with Stattic together with the CDK4/6 inhibitor palbociclib resulted in enhanced tumor cell death upon XVir-N-31 infection (Hindupur et al., 2020). It has been hypothesized that suppressing JAK/STAT signaling could increase OAd replication, and the results confirmed this, showing greater replication and viral particle production with the combination treatment.

### 5.3 Oncolytic adenoviruses and immune checkpoint blockade in kidney and bladder tumors

The discovery of T-cell exhaustion, and its consequent dampening of body's anti-tumor immune response, was a milestone that laid the foundation for immune checkpoint blockade therapy. Previous research suggested that a selective glycogen synthase kinase-3 beta (GSK-3β) inhibitor 9-ING-41 suppresses the expression of immune checkpoint molecules (Shaw et al., 2022). Co-administration of a triple-transgene-armed HY-oAd (Yoon et al., 2024) with 9-ING-41 produces a potent anti-tumor effect in a subcutaneous bladder cancer model. The superior effect of this combination therapy is associated with increased extracellular matrix (ECM) degradation, enhanced apoptosis, greater T-cell accumulation in tumor and spleen tissues, and elevated numbers of CD8^+^PD1^−^ T cells (Yoon et al., 2024). In this strategy, immune checkpoint blockage by 9-ING-41 acts synergistically with the HY-oAd induced immunostimulatory transgenes IL-12 and GM-CSF, along with transgene relaxin-mediated ECM remodeling, to produce a robust anti-tumor response. Similarly, combination treatment with a silk-hydrogel encapsulated Adv-CRB3 (Adv-CRB3@gel, armed with CRB3 and GM-CSF) and an anti PD-L1 antibody significantly reduced tumor size in a subcutaneous bladder cancer mouse model (Zhang et al., 2024b). The synergistic effects of combination therapy surpassed those of the monotherapies, with tumor size remaining stable throughout the experimental period. Analysis of immune cell composition and function within the tumor tissue revealed increased infiltration of CD4^+^ and CD8^+^ T cells and reduced immunosuppressive regulatory T cells (Treg) infiltration. Moreover, expression of functional marker CD25 and CD69 and frequency of IFN-γ^+^ CD8^+^ cells are significantly higher in tumors from the combination-treated group.

Recently, a combination approach with OAds and immune checkpoint inhibitors (ICIs) was evaluated in RCC tumors. This study uniquely used tumor single-cell suspensions derived from patients' clinical samples. Flow-cytometric immune profiling of these samples revealed broad heterogeneity in cell subtypes and immune landscapes. Ad5/3-E2F-d24-HIL7 (TILT-517) was engineered with a chimeric fiber to target renal cancer cells, an E2F promoter driving viral E1A, Δ24 deletion in the E1A gene, Δ145 deletion in the E1B-19K gene, and a hIL-7 transgene. *Ex vivo*, TILT-517 treatment caused more cell killing than either anti-PD-1 or anti-PD-L1 treatment alone. However, no additive effects were observed when TILT-517 was combined with ICIs (Arias et al., 2025). Since the cell suspensions used in the viability assays consisted of mixtures of tumor cells, immune cells, and stromal cells, the lack of observed additive effects was likely due to the MTS assay measuring overall metabolic activity rather than direct tumor cell death. Another contributing factor may be the inherent heterogeneity of the clinical samples. These findings underscore the complexity of translating such approach to the clinic. Further studies were conducted using an HKT-109 syngeneic hamster model with this combination treatment. The tumor growth curves showed improved tumor growth control for TILT-517 plus anti PD-1 treatment; TILT-517 plus anti PD-L1 treatments reached statistically significant improvement compared with monotherapy. Nevertheless, in both *ex vivo* clinical samples and *in vivo* hamster models, TILT-517 treatment increased CD8^+^, CD4^+^, MHCII^+^, and CD51^+^ effector cell populations (Arias et al., 2025). These results highlight the immune-modulation activity of engineered OAds and the potential of this strategy to improve ICIs' clinical outcomes in RCC.

### 5.4 Oncolytic adenoviruses improve the efficacy of CAR-T cell therapy in kidney and bladder tumors

The development of chimeric antigen receptor T cell (CAR-T) therapies has revolutionized the treatment of hematologic malignancies such as leukemia and lymphoma. However, their efficacy in solid tumors remains underwhelming. This is largely due to the lack of universally expressed tumor antigens and the complex TME which restricts CAR-T cell infiltration, proliferation, and function. In this regard, OAds can improve the efficacy of CAR-T therapy by stimulating anti-tumor immune response and remodeling the immunosuppressive TME.

CAR-T therapy has been explored in treating renal cell carcinoma (Mohammadi et al., 2025; Pal et al., 2024; Li et al., 2024a) and bladder cancers (Shen et al., 2024; Zhang et al., 2024a), showing measurable effects but with significant room for improvement in clinical efficacy. An important prerequisite for applying CAR-T therapy in these tumors is the presence of a tumor-specific antigen. Carbonic anhydrase IX (CAIX), a renal carcinoma marker, has been selected to generate CAR-T targeting RCC (CAIX-CAR-T). OAV-DEC is an engineered OAd with an E1B-55K deletion and a decorin transgene, enabling renal tumor cell specific replication and expression of decorin to inhibit TGF-β and other protumor signaling pathways. In a subcutaneous mouse model of human RCC, intratumoral injection of OAV-DEC plus intravenous infusion of CAIX-CAR-T significantly suppressed tumor growth, inhibition rate reaching 90%, whereas sole CAIX-CAR-T treatment achieved only 54% inhibition (Zhang et al., 2022). OAV-DEC treatment markedly increased CAR-T cell numbers in the peripheral blood and enhanced CAR-T infiltration into tumor tissue. Combination treatment also elevated IFN-γ levels in the serum and tumor lysates. Within tumor tissues, mRNA levels of decorin target proteins—including hepatocyte growth factor receptor (Met), VEGF-A, and β-catenin—were significantly reduced. Mechanistically, these OAV-DEC mediated changes reversed EMT, normalized vasculature in the tumor, enhanced antigen presentation, reduced immunosuppressive myeloid-derived cells (MDSCs), and created a favorable chemokine profile for CAIX-CAR-T cell infiltration and function (Zhang et al., 2022). The research group took this strategy further by arming the OAd with transgenes CCL5, and IL-12 (Ad5-ZD55-CCL5-IL12; Fang et al., 2023). Combination treatment of Ad5-ZD55-CCL5-IL12 and CAIX-CAR-T completely eradicated subcutaneous RCC tumors in 80% of mice, a much-improved efficacy than monotherapy. Importantly, mice cured by combination therapy did not develop tumors after re-challenge with tumor cells, indicating that OAds and CAR-T treatment can elicit a potent systemic anti-tumor immunity capable of long-term tumor control.

## 6 Clinical trials evaluating therapeutic oncolytic adenoviruses in kidney and bladder cancers

As discussed above, extensive efforts have been put into developing OAds for treating kidney and bladder cancers, and preclinical studies have demonstrated their strong potential. However, clinical trials are essential to determine their safety and therapeutic benefit in patients. Several OAds have been evaluated in clinical trials for kidney and bladder cancers, as summarized in Table 2.

**Table 2:** Clinical trials evaluating oncolytic adenoviruses for renal and bladder cancers.

**OAds**	**Conditions**	**Phase**	**Combination**	**Route**	**NCT number**	**Trial start**	**References**
CG0070	NMIBC, failed BCG therapy	I		Intravesical	NCT00109655	2005	Burke et al., 2012
CG0070	NMIBC, failed BCG therapy, refuse cystectomy	II		Intravesical	NCT02365818	2015	Packiam et al., 2018
CG0070	NMIBC, failed BCG therapy	II/III		Intravesical	NCT01438112	2014	
CG0070	NMIBC, with CIS +/– Ta/T1, failed BCG therapy	III	n-dodecyl-B-D-maltoside	Intravesical	NCT04452591	2020	Uchio et al., 2022; Leslie, 2023; Tyson et al., 2024
CG0070	NMIBC, with CIS, failed BCG therapy	II	n-dodecyl-B-D-maltoside, Pembrolizumab	Intravesical	NCT04387461	2020	Li et al., 2024b
CG0070	MIBC, cisplatin ineligible	Ib	Nivolumab	Intravesical	NCT04610671	2020	Li et al., 2025
CG0070	NMIBC, BCG-Navie, BCG-exposed or failed BCG therapy	II		Intravesical	NCT06567743	2024	
CG0070	Intermediate risk NMIBC following TURBT	III	n-dodecyl-B-D-maltoside,	Intravesical	NCT06111235	2023	
H101	NMIBC, failed BCG therapy	II	Camrelizumab	Intravesical	NCT05564897	2022	Hua, 2022
ColoAd1	Resectable colon cancer, NSCLC, bladder cancer and renal cell carcinoma	I		Intravenous, intratumoral in colon cancer	NCT02053220	2013	Garcia-Carbonero et al., 2017

BCG, Bacillus Calmette-Guérin; NMIBC, non-muscle-invasive bladder cancer; CIS, carcinoma in situ; MIBC, muscle-invasive bladder cancer; TURBT, transurethral resection of bladder tumor; NSCLC, non–small cell lung cancer.

Among them, CG0070 is one of the most extensively studied, with considerable effort devoted to its development and evaluation across multiple clinical trials. This virus is derived from Ad5 and engineered with an E2F-1 promoter driving E1A expression, and an immune-stimulatory GM-CSF transgene inserted in the E3 gp19KD position under the control of native E3 promoter (Ramesh et al., 2006). Tumor-selectivity is mediated by the E2F-1 promoter-controlled virus replication in Rb-pathway defective tumor cells. Expression of GM-CSF is likewise confined to tumor cells, because the E3 promoter activity depends on the E1A expression. Preclinical studies have demonstrated strong tumor selectivity and potent cytotoxicity of CG0070 in bladder cancer cells and significant anti-tumor efficacy in human bladder tumor mouse models (Ramesh et al., 2006).

The first Phase I trial using CG0070 (NCT00109655) evaluated its safety and dose escalation. A total of 35 patients with NMIBC who had failed BCG treatment received intravesical infusion of CG0070 at different doses, with the highest one of 3 × 10^13^ viral particles. The results showed that intravesical CG0070 was associated with tolerable safety, and the maximum tolerated dose was not reached. Across cohorts, the complete response (CR) rate was 23% in single dose group and 78% in multidose groups, indicating anti-tumor activity against bladder cancer (Burke et al., 2012). Additional Phase II (NCT02365818) and Phase II/III (NCT01438112) trials were conducted to further evaluate the safety and efficacy of CG0070 in patients with high grade NMIBC who had failed BCG treatment. Interim results from trial NCT02365818 reported an overall CR rate of 47% at 6 month and CG0070 remained well tolerated (Packiam et al., 2018). Riding that success, a Phase III trial (NCT04452591) was launched to confirm efficacy of CG0070 in a larger cohort of high-risk BCG-unresponsive NMIBC patients. In this trial, n-Dodecyl-BD-Maltoside (DDM) was instilled as a transduction-enhancing agent (Uchio et al., 2022). A very promising CR rate of 75% was observed, and 97.3% of patients remained free from progression to muscle-invasive bladder cancer (MIBC) at 12 months (Uchio et al., 2022; Leslie, 2023; Tyson et al., 2024). These results could support regulatory filings and broader clinical adoption.

The combination of CG0070 and the anti PD-1 drug pembrolizumab was tested for potential synergistic efficacy in a Phase II trial (NCT04387461). Patients with BCG-unresponsive NMIBC received intravesical instillation of CG0070 and IV injection of pembrolizumab. The CR rate was 82.9% at 3 months and 57.1% at 12 months. No overlapping or synergistic toxicities were observed with the combination therapy compared to individual monotherapy (Li et al., 2024b). In addition, combination therapy with the anti PD-1 agent nivolumab was evaluated in cisplatin-ineligible patients with muscle-invasive bladder cancer (MIBC) in a Phase Ib trial (NCT04610671). In this clinically more serious condition, the combination treatment produced a pathological CR rate of 42.1% and a 1-year recurrence-free survival of 70.4%. The safety profile was favorable, no dose-limiting toxicities (DLTs) and no grade 3 or higher treatment-related adverse events (TRAEs) were encountered (Li et al., 2025). Analyses of patients' urine samples showed increased type-I interferon –induced chemokines, CXCL9 and CXCL11 after CG0070 treatment. Although increased infiltration of CD3^+^CD4^+^ and CD3^+^CD8^+^ T cells was seen in both responders' and non-responders' biopsy samples, expression of CD39, a surrogate marker of tumor-reactive CD8^+^ cells, was elevated only in patients with pathological CR. An increased number of tertiary lymphoid structures (TLSs) after treatment was observed solely in responders. These findings confirm that OAds and ICI treatment can elicit potent cellular and humoral anti-tumor immunity (Li et al., 2025). Investigation of CG0070 continues. A Phase II trial (NCT06567743) extends safety and efficacy evaluation of CG0070 monotherapy to BCG-naïve patients. The most recent registered Phase III trial (NCT06111235) will recruit patients with intermediate risk NMIBC following transurethral resection of bladder tumor (TUBRT) to evaluate recurrence free survival (RFS) with or without adjuvant CG0070 treatment. The expanding clinical scope underscores CG0070's growing potential as a versatile therapy for bladder cancers.

Other OAds that have entered clinical trials include H101, which is genetically modified with a deletion of E1B-55K and part of the E3 region to enhance tumor selectivity. H101 was the first oncolytic virus to receive regulatory approval in China. In 2005, it was approved for advanced head and neck cancer in combination with chemotherapy by Chinese State Food and Drug Administration (Yu and Fang, 2007). Because of its established safety record and immunostimulatory properties, a Phase II trial (NCT05564897) was launched to evaluate the safety and efficacy of H101 combined with the PD-1 inhibitor camrelizumab in BCG-unresponsive NMIBC (Hua, 2022).

Besides oncolytic adenoviral therapy, an adenovirus-based gene therapy for BCG-unresponsive NMIBC has reached a Phase III trial (NCT02773849). Nadofaragene firadenovec (ADSTILADRIN) is a replication-deficient recombinant adenovirus that delivers human IFNα2b gene to the bladder epithelium via intravesical instillation. Five-year follow-up data showed overall survival rates of 80, 76, and 86% in the CIS, Ta and T1 cohorts, respectively (Boorjian et al., 2021; Narayan et al., 2024). This study speaks for the therapeutic potential and durable benefit of adenovirus-based cancer gene therapy in bladder cancer treatment, particularly highlighting the anti-tumor effect of immunostimulatory molecules. The US FDA has approved this adenoviral therapy for adult patients with high-risk NMIBC who are unresponsive to BCG.

At present, clinical trials evaluating the efficacy of OAds in renal cancer are limited. A Phase I mechanism of action (MOA) trial (NCT02053220) enrolled patients with resectable bladder cancer, renal cancer, colorectal cancer (CRC) and non-small cell lung cancer (NSCLC) to assess the feasibility of intravenous delivery of ColoAd1, a chimeric group B Ad11p/Ad3 OAd (Garcia-Carbonero et al., 2017). Overall, these studies have paved the way for the clinical translation of OAds in bladder and renal cancer therapy. The encouraging results position CG0070 as a leading oncolytic viral therapy for bladder cancers.

## 7 Future perspectives

Although accumulated data demonstrates potential utility of OAds for therapy of renal and bladder cancers, several obstacles remain to be addressed before this therapeutic modality may meet with clinical success.

Almost all OAds targeting renal cancers are administered by intratumoral injection. Owing to the anatomic location, OAds for bladder cancers are conveniently delivered by intravesical instillation. These methods could achieve anti-tumor effects in localized tumors; however systemic administration of OAds will be necessary to effectively target metastatic cancers (Atasheva and Shayakhmetov, 2021). Intravenously administered wildtype Advs or Adv vectors encounter numerous neutralizing factors in the blood, including antibodies, complement proteins, and coagulation factors. In addition, Ad5, which is extensively used to build OAds, is largely sequestered by the liver, limiting its systemic bioavailability and contributing to hepatotoxicity (Paolo et al., 2009). The major virus capsid protein, hexon factor X (FX) interaction has been identified as the mechanism facilitating virus sequestration in the liver after intravenous administration (Kalyuzhniy et al., 2008; Waddington et al., 2008). Accordingly, hexon-mutated viruses with ablated FX interaction exhibit dramatically reduced transgene expression in the liver (Waddington et al., 2008). Hexon modification as a liver-detargeting strategy has also been applied in constructing OAds. For example, substitution of the Ad3 hexon protein onto the Ad5 capsid improved antitumor efficacy following systemic administration in a subcutaneous ovarian cancer mouse model (Short et al., 2010). Ad-GL-HB, bearing a modification in hypervariable loop 5 (HVR5) of the hexon, demonstrated enhanced systemic anticancer efficacy and reduced hepatotoxicity in prostate and liver cancer mouse models (Shashkova et al., 2009). These strategies are worth exploring in developing systemically delivered OAds for kidney and bladder cancers, as they could specifically address the high recurrence and metastasis risk usually associated with MIBC and ccRCC.

Pre-existing antiviral immunity represents another major obstacle to the systemic administration of OAds and can also negatively affect OAds delivered through other routes. A high prevalence of anti-Ad5 immunity has been reported in the human population (Chirmule et al., 1999). There is no significant difference in neutralizing antibody (NAb) seroprevalence between healthy adult and cancer patients (71.57 vs. 67.05%; Zhao et al., 2018). Such widespread antiviral immunity severely limits the clinical utility of OAds based on Ad5 serotype. Moreover, Ad5-specific memory T cell and humoral responses have been linked to increased toxicity and raise safety concerns (Zaiss et al., 2009). Strategies to circumvent pre-existing immunity center on modifying the highly immunogenic capsid proteins or replace them with counterparts from rare Adv serotypes. rAd5HVR48(1-7) is a Ad5 based vector with all seven hexon HVRs replaced with the corresponding HVRs from Ad48, a much rarer adenovirus. rAd5HVR48(1-7) carrying simian immunodeficiency virus (SIV) Gag gene produced comparable levels of Gag-specific CD8^+^ T lymphocyte responses in mice even in the presence of high levels of pre-existing anti-Ad5 immunity (Roberts et al., 2006). This example from vaccinating setting suggests that hexon-chimeric vector incorporating rare serotypes can overcome Ab-mediated neutralization, when delivered locally, and this strategy could potentially broaden the applicability of OAds to a larger population of kidney and bladder cancer patients. It remains unclear whether modification of hexon HVRs alone may suffice to avoid OAd neutralization upon intravenous virus administration.

A few studies have explored the use of cell-based carriers loaded with OAds to improve viral delivery to tumor sites. Human bone marrow-derived mesenchymal stem cells (MSCs) are commonly employed as carriers for OAds. In one study, three key features of MSCs have been exploited for RCC-specific delivery and release of OAds: (1) hMSCs migrate toward the chemoattractant PDGF-AA secreted by RCC cells; (2) hMSCs possess an intrinsic osteoblast—commitment property, rendering them capable of Vitamin D3 (VD3)-induced transgene expression; and (3) the oncolytic virus *Ad-hOC*-E1, in which the E1A and E1B genes are controlled by the human osteocalcin promoter, exhibits VD3-dependent viral replication in both hMSCs and RCCs. As a result, intraperitoneal injection of *Ad-hOC*-E1-loaded hMSCs demonstrated the most effective anti-tumor activity in an orthotopic RCC mouse model followed with VD3 treatment (Hsiao et al., 2012).

The tragic death of a participant in the Adv gene-therapy trial in 1999 (Raper et al., 2003) severely hampered the development of Advs for gene therapy, vaccine and oncolytic therapy. This highlights the importance of balancing OAd toxicity reduction and pro-inflammatory cytokine induction for the effective anti-tumor immunity. The severe adverse reaction after the infusion of Ad5-based gene therapy vector occurred quickly, within 18 hours post vector administration, and the patient died due to disseminated intravascular coagulation and multi-organ failure. Although stimulating innate immune response is necessary for OAds to exert anti-tumor effects, exacerbated systemic inflammation after administration of unmodified OAds is detrimental to patients and occurs prior to any anti-tumor benefits can be realized. Modification of Adv capsid can dramatically reduce inflammatory response after intravascular vector administration (Atasheva et al., 2020), and new generation of OAds should combine beneficial modifications of capsid and viral genome to improve safety and efficacy of therapy.

As more data from advanced preclinical models and clinical trials using OAds accumulate, it is expected that adoption of this therapeutic vector platform for treating various types of cancer may occur in the not-so-distant future.
